# Combining Coagulation and Electrocoagulation with UVA-LED Photo-Fenton to Improve the Efficiency and Reduce the Cost of Mature Landfill Leachate Treatment

**DOI:** 10.3390/molecules26216425

**Published:** 2021-10-25

**Authors:** Javier Tejera, Daphne Hermosilla, Antonio Gascó, Carlos Negro, Ángeles Blanco

**Affiliations:** 1 Department of Chemical Engineering and Materials, Chemistry Science Faculty, Complutense University of Madrid, 28040 Madrid, Spain; jttejo@ucm.es (J.T.); ablanco@ucm.es (Á.B.); 2 Department of Forest and Environmental Engineering and Management, E.T.S.I. Montes, Forestal y del Medio Natural, Universidad Politécnica de Madrid, 28040 Madrid, Spain; daphne.hermosilla@upm.es (D.H.); antonio.gasco@upm.es (A.G.); 3 Department of Agricultural and Forest Engineering, EIFAB, Campus Duques de Soria, University of Valladolid, 42005 Soria, Spain

**Keywords:** wastewater treatment, recalcitrant compounds, sustainable technologies, electrocoagulation, landfill leachate, bio-degradability enhancement

## Abstract

This study focused on the reduction of the treatment cost of mature landfill leachate (LL) by enhancing the coagulation pre-treatment before a UVA-LED photo-Fenton process. A more efficient advanced coagulation pretreatment was designed by combining conventional coagulation (CC) and electro-coagulation (EC). Regardless of the order in which the two coagulations were applied, the combination achieved more than 73% color removal, 80% COD removal, and 27% SUVA removal. However, the coagulation order had a great influence on both final pH and total dissolved iron, which were key parameters for the UVA-LED photo-Fenton post-treatment. CC (pH = 5; 2 g L^−1^ of FeCl_3_6H_2_O) followed by EC (pH = 5; 10 mA cm^−2^) resulted in a pH of 6.4 and 100 mg L^−1^ of dissolved iron, whereas EC (pH = 4; 10 mA cm^−2^) followed by CC (pH = 6; 1 g L^−1^ FeCl_3_6H_2_O) led to a final pH of 3.4 and 210 mg L^−1^ dissolved iron. This last combination was therefore considered better for the posterior photo-Fenton treatment. Results at the best cost-efficient [H_2_O_2_]:COD ratio of 1.063 showed a high treatment efficiency, namely the removal of 99% of the color, 89% of the COD, and 60% of the SUVA. Conductivity was reduced by 17%, and biodegradability increased to BOD_5_:COD = 0.40. With this proposed treatment, a final COD of only 453 mg O_2_ L^−1^ was obtained at a treatment cost of EUR 3.42 kg COD^−1^.

## 1. Introduction

The generation of municipal solid waste is becoming a key challenge due to population and economic growth as well as other changes in our lifestyle [1]. Kaza et al. [2] estimated a 70% increase of the annual world waste generation, from 2.0 to 3.4 billion tons, for 2050. Notwithstanding the huge area that requires to be implemented and the high environmental and health hazards that it may cause because of the generation of a great amount of leachate, landfilling is the most common option applied worldwide to manage this type of waste due to its low cost and relative simplicity of operation [3]. Although incineration can reduce the volume of the disposed waste and complementary produce energy [4,5], this practice is not widely used, and it still produces 10–20% of ashes to be landfilled [6].

Landfill leachate (LL) is a very harmful wastewater that is generated by the percolation of rainwater through landfilled waste, where different aerobic and anaerobic degradation [7] and physico-chemical decomposition [8] processes occur; thus, LL needs to be adequately treated to avoid negative environmental impacts. LL has different characteristics depending, for example, on landfill age, origin of the waste, and climate conditions [9]. The most important characteristics to consider for classifying LL are: pH, conductivity, ammonia nitrogen content, chemical oxygen demand (COD), biochemical oxygen demand (BOD_5_), and biodegradability, expressed as the ratio of BOD_5_:COD. These factors are highly affected by the age of the landfill [10]. Whereas young LL (<10 years) presents a high biodegradable organic load (COD > 4000 mg O_2_ L^−1^, BOD_5_:COD ≈ 0.5, ammonia nitrogen < 400 mg L^−1^, and pH ≈ 6.5), mature LL (>20 years) holds low biodegradable organic load (COD < 4000 mg L^−1^, BOD_5_:COD < 0.1, ammonia nitrogen concentration > 400 mg L^−1^, and pH ≈ 7.5) [11,12,13].

Biological processes are a good option for the treatment of young LL, but they are inefficient for mature LL, which must be treated by a combination of physical and chemical processes [11,14,15,16]. Although this is an old problem, there is not an accepted best treatment strategy for mature LL yet. Several authors [17,18,19] proposed the application of coagulation followed by photo-Fenton treatment, which achieved efficiencies of 60–89% depending on the coagulant used (polyaluminum chloride or ferric chloride) and the pH. Coagulation is based on the removal of organic matter by destabilization of colloids and particles due to charge neutralization, which produces their aggregation and facilitates their separation by sedimentation or flotation [20]. This process is carried out by adding the coagulant dosage required to neutralize the charge of the contained particles in the wastewater. Therefore, this treatment requires high coagulant dosages that increase both the conductivity and the inorganic pollution of the treated effluent due to the addition of counterions, such as chloride, to the media.

Complementarily, the Fenton process is based on quick and non-selective oxidation of recalcitrant organic matter through the action of a hydroxyl radical. Hydroxyl radicals are formed by the homolytic rupture of H_2_O_2_ by Fe^2+^ under acidic conditions [21]. The Fenton process is recently gathering relevance because of its simple operation and potential application to the direct mineralization of organic pollutants and the improvement of biodegradability by partial oxidation, at least. To enhance the catalytic capacity, increase treatment efficiency, and reduce iron sludge generation, photo-Fenton processes are an optimal alternative. Radiation accelerates the reduction of Fe^3+^ to Fe^2+^ and increases the destruction rate of organic pollutants. The key is the reduction of metal catalysts by photochemical processes [22]. These advanced oxidation processes (AOPs) have widely been investigated for LL treatment, and it was already reported that its combination with other technologies is highly convenient to develop optimized efficient and cost-effective wastewater treatment strategies [15].

To avoid the addition of counterions, some authors proposed the use of electrocoagulation (EC) in combination with sonication, ozone, biological treatment, or electro-oxidation for the treatment of LL [23,24,25]. EC is versatile, simple in operation, and can handle a wide range of pollutants. It is based on the same principle as coagulation, the destabilization of contaminants, with the difference that the coagulant is generated in situ by electro-dissolution of sacrificeable metal electrodes [26]. Asaithambi et al. [23] reported 100% color and 97.5% COD removals when the combination of sonication, ozone, and EC was applied for the treatment of LL, but this required 6 g L^−1^ of NaCl to be added to the media to improve the efficiency and reduce the energy consumption of the process. Djelal et al. [17] studied the combination of EC with a biological treatment, achieving 33% and 56% of COD removal when 23 or 98 A m^−2^ were respectively applied to mature LL. Thanh Le and Khai Cao Le [25] achieved a 95% COD removal training a process with EC followed by electro-oxidation. However, in this case, the promotion of free chlorine formation was an important drawback to the application of this treatment alternative.

Precisely, the combination of EC and conventional coagulation (CC) has not been assessed yet, although it might be a good alternative to achieve high pre-treatment efficiency before an AOP without the drawback of the addition of counterions in the case of CC standalone or the high power consumption of a single EC pre-treatment. This combination was previously used for the successful pre-treatment of palm oil effluent [27], slaughter house wastewater [28], and textile wastewater [29], but there are no references to the application of this coagulation combination as pre-treatment for LL depuration before an oxidation treatment. Furthermore, there might be further advantages if the coagulant used in both coagulation pre-treatments is iron, as the remaining iron content may serve as the catalyst in a posterior photo-Fenton treatment. In short, the hypothesis that an enhanced coagulation by the combination of coagulation pre-treatments before an optimized photo-Fenton treatment might be more efficient in removing the contaminant load at a lower or similar total cost deserves investigation. Therefore, the main objective of this study was to assess the synergic effects of the combination of EC and CC as pre-treatments of a UVA-LED photo-Fenton treatment of mature LL in terms of treatment efficiency and cost that might provide a potential feasible LL treatment strategy to be considered for full-scale applications.

## 2. Materials and Methods

### 2.1. Chemicals

Ferric chloride FeCl_3_∙6H_2_O (99%) was used as the coagulant agent as a 69% (wt%) solution in distilled water. Hydrogen peroxide (35%, wt%) was used in the Fenton processes. Sulfuric acid (H_2_SO_4_, 96–98%) was used for pH adjustment. All chemicals were purchased from Sigma-Aldrich (San Luis, Missouri, USA). An anionic flocculant of high molecular weight was supplied by Kemira (Helsinki, Finland) and was used as a 0.025% (wt%) solution in distilled water.

### 2.2. Landfill Leachate (LL)

A mature LL with very low biodegradability (BOD_5_:COD = 0.01) and a COD = 5025 mg O_2_ L^−1^ was collected from a municipal solid waste landfill located in Golmayo (Soria, Spain), which began to operate in 1997. Table 1 shows the main characteristics of the sampled mature LL.

### 2.3. Conventional Coagulation Followed by Electrocoagulation

Current density optimization was carried out on a CC-treated LL for which the best CC conditions reported in the literature were used (initial pH = 5, and 2 g L^−1^ of FeCl_3_6H_2_O + 10 mg L^−1^ of anionic flocculant were added) [11].

The EC pretreatment of the CC pre-coagulated LL was carried out using two iron electrodes (anode and cathode) of 20 × 5 cm in total that were submerged 50 cm^2^ and separated by 3 cm. The amount of dissolved iron after EC was measured by atomic absorption and verified by weighing the anode. Experiments were carried out using 500 mL samples that were magnetically stirred at 100 rpm at room temperature, and current intensity was fixed with a GLPS 3010 power supply (0–30 V and 0–10 A) from Geti (the Czech Republic); a current density range of 5–20 mA cm^−2^ was tested.

### 2.4. Electrocoagulation Followed by Conventional Coagulation

The EC treatment was carried out at the best process conditions that were reported in a previous study (initial pH = 4, 10 mA cm^−2^, and 3 cm of distance between iron electrodes) [30].

CC was then carried out in a 500 mL beaker containing 250 mL of pre-electro-coagulated LL. Coagulation was carried out by a jar test using a ferric chloride concentration between 1–5 g L^−1^ at different pH values (4–8). Ferric chloride was added and agitated for 5 min at 150 rpm. Then, the anionic flocculant was added (10 mg L^−1^), and the samples were agitated for 30 min at 50 rpm. Afterwards, the samples were left to settle for sedimentation for 60 min.

### 2.5. UVA-LED Photo-Fenton

UVA-LED photo-Fenton experiments were carried out on the best dual pre-treated LL (EC followed by CC, as it is shown in the Results and Discussion section) using the residual iron left in the media after the pre-treatment. The initial pH of the pre-treated LL was adjusted to 3, and the experiments were carried out at room temperature. The LED photo-Fenton treatment was evaluated by studying three different values for the [H_2_O_2_]:COD concentration ratios, namely: 2.125, 1.063, and 0.531 (the stoichiometric value, its half, and a quarter of it, respectively). These ratios were calculated as follows [31]: 1 g COD → 1 g O_2_ → 0.03125 mol O_2_ → 0.0625 mol H_2_ O_2_ → 2.125 g H_2_O_2_.

**Figure 1:**
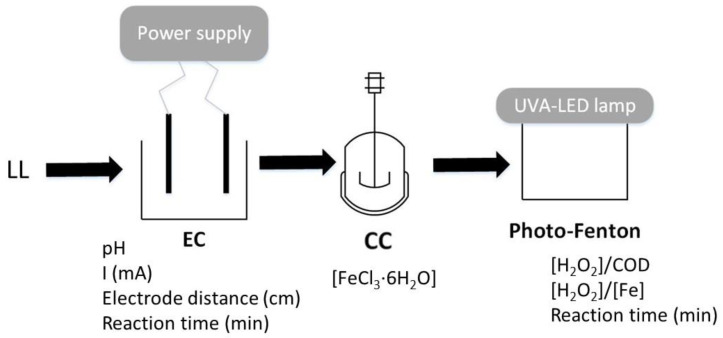
Schematic representation of the full optimized treatment strategy.

The set-up used for the UVA-LED photo-Fenton consisted in a 9 cm diameter reactor filled with 100 mL of pre-treated LL. This was magnetically stirred and irradiated with a 4 W UVA LED lamp made of 10 LED emitters of 367 nm (CUN6GB1A, Seoul Viosys, Asan, North Korea) uniformly disposed with a total photon flux of 4.15 · 10^20^ photon s^−1^ m^−2^ (measured by potassium ferrioxalate actinometry [32,33]) generated by the application of 125 mA of current intensity. The LED lamp was located at 4.5 cm from the LL surface. A schematic representation of the full optimized treatment strategy is shown in Figure 1.

### 2.6. Analytical Determinations

All analyses were performed according to the Standard Methods for the Examination of Water and Wastewater [34].

Conductivity and pH were measured using a Sension^TM^ + MM374 pH-meter (Hach, CO, USA) equipped with pH and conductivity probes. COD was measured following the Nanocolor^®^ test method (Macherey-Nagel GmbH, Düren, Germany) using an Aquamate UV-Vis spectrophotometer (Thermo Fisher Scientific, WA, USA) to perform the measurements. BOD_5_ was determined following Standard Method 5210 B. Total dissolved iron was measured by atomic absorption spectrometry (3111 B, 3111 E) with a Varian SpectrAA 220 spectrophotometer (Varian, CA, USA). Iron (II) was measured by the phenanthroline method. Total organic carbon (TOC) and total bound nitrogen (TN_b_) were determined by the combustion-infrared method using a Multi N/C^®^ 3100 TOC/TN analyzer (Analytik Jena AG, Jena, Germany) with catalytic oxidation on cerium oxide at 850 °C. UV-254 absorbance was measured using a Varyan Cary 50 UV-visible spectrophotometer (Varian, CA, USA) using 1 cm pathway quartz cuvettes (Hellma, Müllheim, Germany). Specific UV absorbance (SUVA) was calculated as SUVA = 100·(UV-254)/TOC, being UV-254 the absorbance of the sample at this wavelength per cm (cm^−1^), which provides a reference for the concentration of organic matter holding aromatic rings or unsaturated bonds in their molecular structures. Color was determined by measuring absorbance at 405 nm using a filter photometer (PF-11 from Macherey-Nagel, Düren, Germany). H_2_O_2_ concentration was determined by the titanium sulfate spectrophotometric method [35].

## 3. Results and Discussion

### 3.1. Electrocoagulation of Conventionally Pre-Coagulated LL

**Figure 2:**
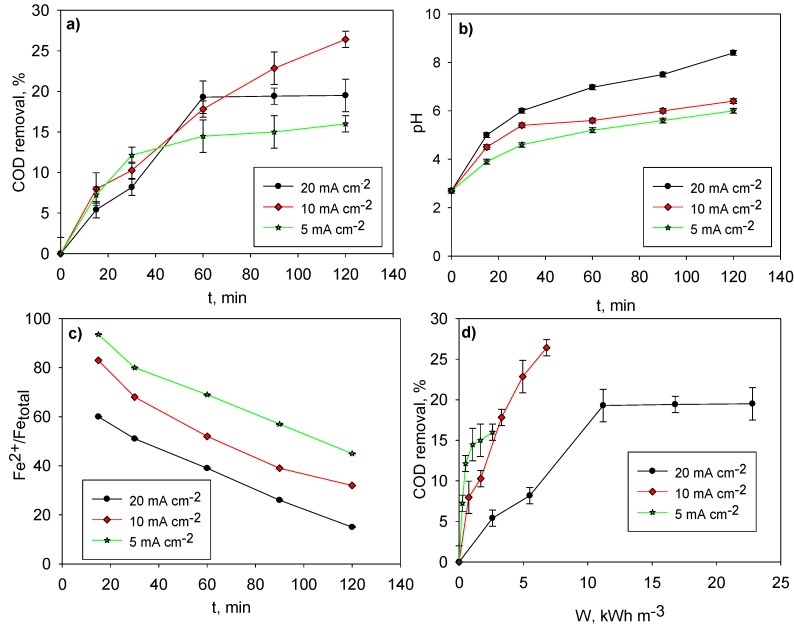
Current density optimization for the electro-coagulation treatment (at 3 cm of electrodes distance) of conventionally coagulated landfill leachate (at pH = 5 and 2 g L^−1^ of FeCl_3_ 6H_2_O): (**a**) COD removal evolution along treatment; (**b**) evolution of pH along treatment; (**c**) distribution of iron species during the process; and (**d**) COD removal vs. power consumption.

The CC pre-treated LL with a COD of 1358 mg O_2_ L^−1^ and pH = 2.4 was treated by EC. As expected, COD removal increased with current density. In a 2 h treatment at a current density of 5 mA cm^−2^, an extra 16% COD removal was achieved, which increased up to 26% when current density was doubled (Figure 2a). However, when current density was increased to 20 mA cm^−2^, a 20% COD removal was addressed. This could be explained by the fast increase of the pH that occurs during the EC treatment at this current density value (Figure 2b) because optimal coagulation would be performed at pH = 4–5, and higher pH values would be detrimental [11]. The initial pH = 2.4 increased to 7.5 and 8.4 after 1 and 2 h of treatment, respectively, at a current density of 20 mA cm^−2^; whereas the final pH after 2 h of treatment was just 6.4 at 10 mA cm^−2^. A current density of 5 mA cm^−2^ produced a slower increment of pH than for the current density of 20 mA cm^−2^. In short, the pH increased as the result of the following reaction, which was produced in the cathode: 2 H_2_O + 2 e^−^ → H_2_ + 2 ^−^OH. Additionally, the generation of ^−^OH was faster and more intense as higher values of current density were applied.

It should be noted that the first species formed during EC was Fe^2+^, which was oxidized to Fe^3+^ thanks to the application of current density, and it was Fe^3+^ which was actually implicated in removing the major fraction of the COD during the coagulation process. As pH was increasingly pushed above the optimal value of 4–5 for Fe^3+^ coagulation along the processes, and as current density was pushed higher as well, the ratio Fe^2+^:Fe_total_ correspondingly decreased (Figure 2b,c). Considering all the above, it finally resulted that the overall removal of the COD was optimized for a current density of 10 mA cm^−2^ (>25%), whereas it was lower for either 5 or 20 mA cm^−2^ (≈15–20 %) (Figure 2a–d) because the current density value was too low in the first case and because the pH increased too much above optimal values of coagulation performance in the second one.

The current density of 20 mA cm^−2^ consumed 22.8 kWh m^−3^ (Figure 2d), whereas a current density of 10 mA cm^−2^ decreased power consumption to 6.8 kWh m^−3^ and achieved a higher COD removal, as commented above. The current density of 5 mA cm^−2^ consumed 2.6 kwh m^−3^, but the lowest COD removal result was obtained. These power consumption results were also directly proportional to the applied voltage along each trial, which remained more or less constant and proportional to each current density value, provided the conductivity of the media did not change much along the processes. In addition, the total dissolved iron content after a 10 mA cm^−2^ treatment was 100 mg L^−1^, whereas it was only 60 mg L^−1^ after the 5 mA cm^−2^ EC pre-treatment, and it was 150 mg L^−1^ after the EC treatment at 20 mA cm^−2^. This is important because the next step of the treatment was a UVA-LED photo-Fenton process, where the kinetics were directly related to the content of dissolved iron in the solution. Therefore, the selection of the optimal pre-treatment was necessarily based on the overall treatment cost.

### 3.2. Conventional Coagulation of Pre-Electrocoagulated LL

**Figure 3:**
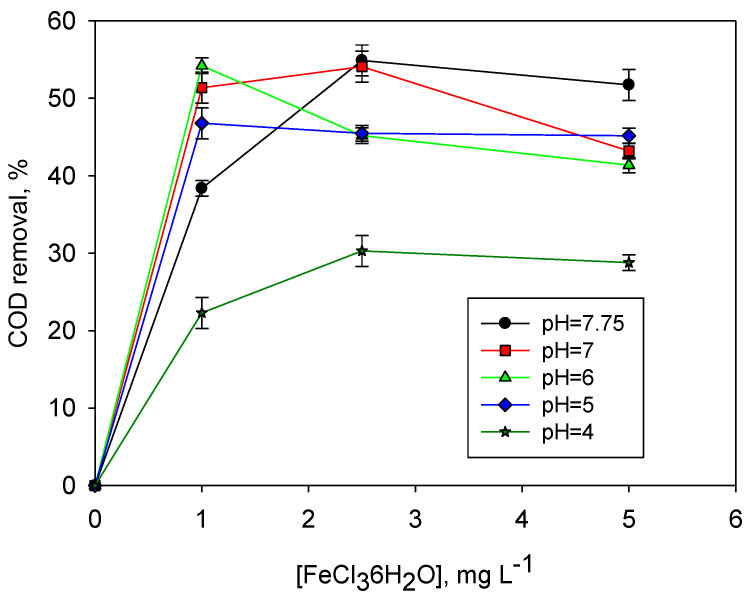
COD removal at different initial pH values for the conventional coagulation treatment (different coagulant dosages) of pre-electrocoagulated LL (pH = 4, 10 mA cm^−2^, 3 cm electrode distance).

The electrocoagulated LL had a slightly higher COD than the coagulated LL, with a COD value of 1856 mg O_2_ L^−1^ and a pH = 7.75. Figure 2 presents the results of the EC followed by CC trials. In general, the required concentration of the coagulant (FeCl_3_∙6H_2_O) to obtain better COD removal results decreased when the initial pH was adjusted to more acidic values before the CC stage. The two highest tested initial pH values (7.8 and 7.0) achieved an extra COD removal of 54% at a dose of 2.5 g L^−1^. Reducing the pH to 6 before CC resulted in the same 54% COD removal at the lower dosage of 1 g L^−1^. However, if the pH was further reduced, COD removal decreased to 46% for an initial pH = 5 and 1 g L^−1^ of coagulant and even down to 30% for an initial pH = 4 and a dose of 2.5 g L^−1^ (Figure 3). This effect was the result of the acidification of the medium that occurred when the coagulant was added. In a first step of coagulation, Fe^3+^ was hydrolyzed capturing ^−^OH, as expressed by the following overall equation: FeCl_3_ + 3H_2_O → Fe(OH)_3_ + 3HCl; thus, a subsequent reduction of the pH was produced.

In addition, most of the alkalinity was removed because of the pH adjustment that was performed before the EC step. As a result, a little addition of coagulant led the pH to decrease below 4. As the optimal pH for ferric chloride CC is about 4–5, lower pH values led to a lower efficiency of the process.

In summary, results show that the best conditions for the CC of pre-electro-coagulated LL were an initial pH = 6 and the addition of 1 g L^−1^ of FeCl_3_∙6H_2_O. The lower FeCl_3_∙6H_2_O addition implied lower chloride addition as well, and reducing this contaminant was important because of its major impact on the Fenton reaction [36] compared to the increase of the presence of sulfate, which was derived from the need to adjust the pH.

### 3.3. Comparison of CC-EC and EC-CC Combinations

It can be noticed that, when EC was followed by CC, results were slightly better in terms of COD, SUVA, color, and conductivity removals (Table 2) than the CC + EC pre-treatment alternative. An increase in the pretreatment efficiency may have led to a significant cost reduction in the subsequent UVA-LED photo-Fenton treatment; however, the synergic effect of pH and total dissolved iron also had to be considered due to its relevance for the photo-Fenton process kinetics, which was the key to reduce the power consumption of this process [37].

Fenton processes are favored at acidic pH [38] because Fe^2+^, which is responsible for the homolytic splitting of hydrogen peroxide, is predominant. In addition, the higher iron concentration presence after the EC + CC case may have also led to a faster Fenton reaction. In summary, CC followed by EC reached a final pH = 6.4 and a final total dissolved iron content of 100 mg L^−1^, whereas EC followed by CC ended in a pH = 3.4 and 210 mg L^−1^ of dissolved iron (Table 2), which were significantly better initial conditions to perform a more efficient posterior UVA-LED photo-Fenton treatment.

The obtained results, therefore, showed a better synergic effect of both coagulation processes when EC was run before CC. In comparison with current results, Tejera et al. [11] reported a lower 68% COD removal when only CC was used as a pre-treatment (compared with the higher figures included in Table 2 for the herein assessed EC + CC and CC + EC combinations), and an inconvenient increment of electric conductivity up to 22.5 mS cm^−1^ (+12.4%) was addressed as well. Another study only considering EC as pretreatment [30] also showed a lower 62% COD removal, but conductivity was reduced 24%. In addition, Dia et al. [39] addressed another lower 65% COD removal in the treatment of pre-biofiltrated LL with single EC at a current density of 10 mA cm^−2^. However, with the herein proposed dual coagulation combination pre-treatment strategy, the overall COD removal was higher than 80%, which represents a significant improvement.

In short, it can be concluded that EC (pH = 5, 10 mA cm^−2^, and 3 cm of electrode distance) followed by CC (initial pH = 6 + 1 g L^−1^ of FeCl_3_∙6H_2_O) is the best assayed pre-treatment combination before a UVA-LED photo-Fenton process. On one hand, the efficiency of Fenton processes in targeting organic pollutants can be significantly enhanced thanks to the benefit of reducing the content of suspended solids, colloids, and color, because these compounds may act as photon absorbers and hydroxyl radical scavengers [40]. On the other hand, this pre-treatment led to a final acid pH value of 3.4 (very close to an optimal pH = 2.8 for the Fenton treatment of LL [37]) and left enough iron content in the effluent to avoid adding further dosage of chemicals aiming to enhance the oxidation treatment step. In comparison, a final pH = 2.8 after a CC pre-treatment was achieved in a previous study [11] and, in another work [30], it was required to adjust the pH after an EC pre-treatment (thus incrementing the sulfate content) before performing the photo-Fenton treatment of a similar LL. In both cases, worse COD removal efficiencies were reported (68% and 62%, respectively). Therefore, this combined coagulation strategy represents a relevant enhancement alternative for the treatment of mature LL.

### 3.4. UVA-LED Photo-Fenton

The UVA-LED photo-Fenton experiments were carried out at the final pH value resulting from the EC + CC dual pre-treatment discussed above (pH = 3.4). Additionally, they were performed with the amount of dissolved iron remaining in the solution after this step (210 mg L^−1^), avoiding the addition of more iron from an external source. Thus, the combination of EC with Fenton processes, which was previously not considered feasible as a standalone option [26], may result in a more attractive treatment alternative for LL when it is appropriately combined with CC.

**Figure 4:**
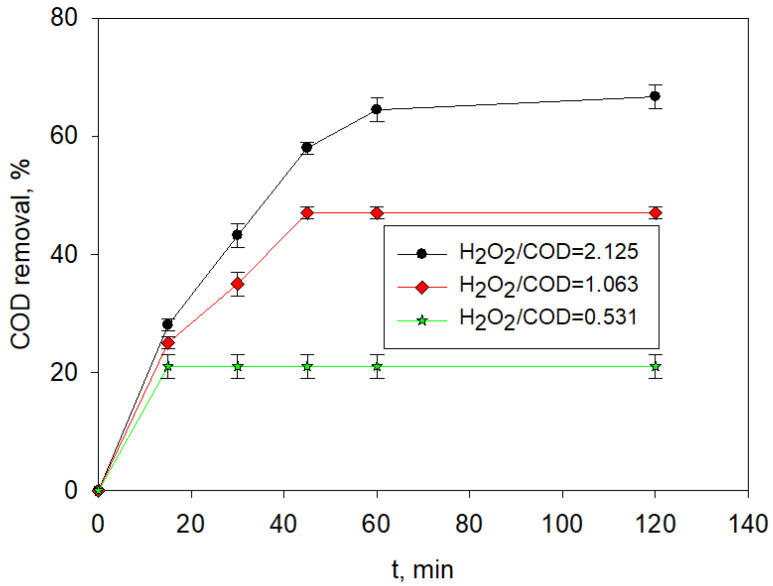
COD removal along the UVA-LED photo-Fenton treatment (considering different [H_2_O_2_]:COD ratios) of mature LL pre-treated by an optimized electro-coagulation followed by a conventional coagulation CC pretreatment combination.

The COD removals achieved in this step were 66%, 47%, and 21% for the UVA-LED photo-Fenton treatment performed using [H_2_O_2_]:COD concentration ratios of 2.125, 1.063, and 0.531, respectively, and the times required for such treatment results were 120, 45, and 15 min, respectively (Figure 4). Obviously, a lower H_2_O_2_ addition limited the COD removal result, and the duration of the reaction was shorter because the added H_2_O_2_ was totally consumed more quickly.

Considering the [H_2_O_2_]:COD = 2.125 and a 120 min long UVA-LED photo-Fenton treatment of the dual EC + CC pre-treated LL, the overall treatment results addressed 99%, 94%, 67%, and 15% reductions of color, COD, SUVA, and conductivity, respectively, with an increase of the BOD_5_:COD ratio up to 0.45, as it is shown in Table 3. As was recently reviewed [14], a 94% COD removal was only reported to be achieved by the combination of nanofiltration or ultrafiltration with adsorption.

In short, both configurations of the UVA-LED photo Fenton treatment achieved BOD_5_:COD values higher than 0.40 and final COD figures below 500 mg O_2_ L^−1^, indicating that biological or algae post-treatment might be possible if necessary [41]. This final COD value was reported to be below the discharge limits into public sewage networks [40], but it would depend on every local legislation development.

In addition, 45 min of treatment addressed the removal of 99% of the color, 89% of the COD, and 60% of the SUVA when an [H_2_O_2_]:COD = 1.063 was used, and conductivity was reduced to 16.70 mS cm^−1^ (−17%), whereas biodegradability was increased up to BOD_5_:COD = 0.40. Although the higher [H_2_O_2_]:COD ratio of 2.125 gave slightly better removal percentages of COD, color, and SUVA, results at a ratio of 1.063 obtained an interesting COD removal requiring half the H_2_O_2_ addition. Thus, it represents a major reduction of the overall process cost because the treatment is more efficient per unit of added H_2_O_2_. As expected, the photo-Fenton step had no significant effect on conductivity (comparison of Table 2 and Table 3). In a case where the iron residual content must be removed before discharge, an additional precipitation step might be included to comply with the legislation in force.

The optimized treatment strategy addressed 63% COD removal after the EC pre-treatment, which was increased up to 83% after the CC pre-treatment was optimized. Finally, total COD removal figures of 94% and 89% were reported after the UVA-LED photo-Fenton treatment was applied at [H_2_O_2_]:COD ratios of 2.125 and 1.063, respectively.

### 3.5. Treatment Cost

Power consumption, chemicals addition, the use of iron electrodes, and waste management were considered to calculate a first approximation to the treatment cost (Table 4). The price for concentrated sulfuric acid (EUR 130 ton^−1^), iron (EUR 500 ton^−1^), ferric chloride (EUR 200 ton^−1^, 40 wt%), and hydrogen peroxide (EUR 350 ton^−1^, 35 wt%) were obtained (at industrial grade) from www.alibaba.com. Energy (EUR 0.11 kwh^−1^) and sludge management (EUR 0.02 kg^−1^) costs were taken from current average cost figures in Spain. Finally, if a potential additional posttreatment of iron precipitation is necessary to comply with the legislation in force to discharge to the sewer network when other recovery or recycling operations are not considered, approximately EUR 0.2 m^−3^ additional cost must be added into Table 4, as it was estimated from a previous similar research essay [42].

The treatment cost of the direct application of a UVA-LED photo-Fenton treatment without coagulation pretreatment, carried out as a reference at pH = 3 and at the same [H_2_O_2_]:Fe ratio used in the herein proposed EC + CC + UVA photo-Fenton treatment, resulted in 4.77 and EUR 3.83 kg^−1^ of removed COD at the ratios of [H_2_O_2_]:COD = 2.125 and 1.063, respectively. These values are similar to the estimations for the improved treatment with coagulation pre-treatments, namely: 5.05 and EUR 3.42 kg^−1^ of removed COD (Table 4), respectively. The COD removal efficiencies achieved by this standalone UVA-LED photo-Fenton application were 74% and 60% for the [H_2_O_2_]:COD ratio values of 2.125 and 1.063, respectively. These efficiencies were 20–30% lower than the results achieved by the EC + CC + Photo-Fenton strategy.

Comparing both assessed [H_2_O_2_]:COD ratios (2.125 and 1.063) to perform the UVA-LED photo-Fenton treatment, it is worth noticing that the 1.063 [H_2_O_2_]:COD ratio resulted in much more efficient figures in terms of cost per removed kg of COD or m^3^ of treated LL (Table 4). In particular, the cost of the oxidation step itself was estimated about 50% cheaper when the 1.063 ratio was applied because half the [H_2_O_2_] and even lower power use were required in a shorter treatment (45 vs. 120 min). Consequently, the overall cost including the EC + CC separation pre-treatment resulted in EUR 3.42 vs. EUR 5.05 kg^−1^ of removed COD and EUR 15.65 vs. EUR 22.10 m^−3^ of LL for the cases applying [H_2_O_2_]:COD ratios of 1.063 or 2.125, respectively. The achieved values of both treatment configurations were not too different, namely: 453 vs. 293 mg O_2_ L^−1^ of COD; 1.25 vs. 1.05 L mg^−1^ m^−1^ of SUVA; and a BOD_5_:COD ratio of 0.40 vs. 0.45 for the [H_2_O_2_]:COD ratios of 1.063 and 2.125, respectively.

The herein proposed EC + CC + UVA-LED photo-Fenton treatment strategy significantly improved the efficiency of previously reported studies. In particular, Tejera et al. [11] reported similar treatment cost (Table 5) for the treatment of a similar mature LL with the combination of CC and a photo-Fenton process using conventional mercury immersion lamps at the [H_2_O_2_]:COD ratio of 1.063, but significantly worse final results were reported (48% COD removal, conductivity increased about 25%, and biodegradability was just improved up to BOD_5_:COD = 0.32). In addition, LED lamps are potentially less hazardous to dispose of at the end of their life cycle than mercury vapor immersion lamps.

Moreover, in another recent study in which sludge management was not included, we estimated costs of EUR 1.09 and 3.48 per kg of removed COD in the treatment of mature LL consisting of an EC pre-treatment followed by either conventional Fenton or UVA-LED photo-Fenton, respectively, both using [H_2_O_2_]:COD = 1.063 [30]. The herein assessed improved EC + CC + UVA-LED photo-Fenton treatment alternative addressed an estimated cost (without sludge management) of EUR 1.57 kg^−1^ of removed COD but achieved a much higher COD removal. A similar treatment cost of EUR 1.56 kg^−1^ of removed COD was achieved in a previous study [11], but the final COD was twice the final COD of the present study.

In short, Table 5 shows a summary of the comparison of the mentioned treatment alternatives in terms of treatment cost and final COD, addressing that the herein assessed alternative based on EC + CC + UVA-LED photo-Fenton led to a much lower final COD value of the treated LL. All these economic assessments were estimated based on laboratory scale data, that is, just considering the costs of reactants and power consumption. Hence, the lower cost estimation that was reported for the conventional Fenton treatment combination did not account for the feasibility issues of requiring a huge space and a long time of treatment (24 h vs. 45 min in the case of photo-Fenton) [30]. Additionally, treatment results in terms of the removal of COD and SUVA as well as the improvement of biodegradability were better in the herein assessed EC + CC + UVA-LED photo-Fenton treatment strategy. Furthermore, the total cost of such treatment combination is expected to be reduced when implemented at full scale thanks to potential further optimization in the use of chemicals and energy [43].

## 4. Conclusions

New standards have created a great challenge for LL treatment, which needs to be highly efficient at a feasible cost to be industrially applied. The combination of separation and degradation technologies is required for the treatment of mature LL because of the complexity of this type of wastewater, but cost-efficient strategies have to be developed for real industrial applications.

Coagulation combined with a photo-Fenton process is technically viable, but the cost has been reported to be still high. However, based on the herein achieved results, it is concluded that an enhanced coagulation set-up achieved by the optimal combination of EC and CC significantly reduced treatment cost by 55%. This enhanced coagulation pre-treatment together with optimized UVA-LED photo-Fenton treatment of mature LL led to a better overall efficiency (up to 94% COD removal and 17% conductivity reduction for the ratio [H_2_O_2_]:COD = 2.125) at a lower cost than other treatment alternatives. This high performance is comparable to the use of membrane technologies.

The pre-treatment strategy based on CC followed by EC addressed worse overall results than EC followed by CC before performing the UVA LED photo-Fenton process.

EC + CC + UVA-LED photo-Fenton performed at an [H_2_O_2_]:COD ratio of 1.063 was herein reported as an optimal technical and economical treatment for mature leachate. Under these conditions, it was possible to remove 99% of LL color, 89% of its COD, and 60% of SUVA; biodegradability was enhanced up to BOD_5_:COD = 0.40, and conductivity was reduced by a 17%. In this case, the preliminary estimation of the overall treatment cost at laboratory scale considering energy and chemicals use as well as sludge management costs resulted in EUR 3.42 per kg of removed COD.

## Figures and Tables

**Table 1:** Landfill leachate characteristics.

Parameter (LL)	Value
pH	8.2 ± 0.1
Conductivity, mS cm^–1^	20.1 ± 0.8
[Cl^−^], mg L^–1^	2800 ± 200
UV-254, cm^–1^	60 ± 1
Color, mg Pt L^−1^	19,600 ± 1200
COD, mg O_2_ L^−1^	5025 ± 500
BOD_5_, mg O_2_ L^−1^	50 ± 10
BOD_5_/COD	0.01 ± 0.01
TOC, mg C L^−1^	1900 ± 50
SUVA, L mg^−1^ m^−1^	3.2 ± 0.4
TSS, mg L^−1^	1250 ± 50
NH_4_^+^, mg L^−1^	1500 ± 100
TN_b_, mg N L^−1^	1680 ± 50

SUVA (specific ultra-violet absorbance) = (100·(UV-254)/TOC.

**Table 2:** Results of both combinations of coagulation pre-treatments (conventional followed by electro-coagulation and vice versa).

	CC (pH = 5.0 + 2 g L^−1^ FeCl_3_6H_2_O) + EC (pH = 2.4, 10 mA cm^−2^, 3 cm of Electrode Distance)	EC (pH = 5.0; 10 mA cm^−2^; 3 cm of Electrode Distance) + CC (pH = 6 + 1 g L^−1^ FeCl_3_6H_2_O)
pH	6.4 ± 0.1	3.4 ± 0.1
Conductivity mS cm^−1^	16.9 ± 0.8 (16%) *	16.5 ± 0.8 (18%)
Color mg Pt L^−1^	5300 ± 500 (73%)	4900 ± 500 (75%)
COD mg O_2_ L^−1^	1005 ± 90 (80%)	854 ± 70 (83%)
SUVA L mg^−1^ m^−1^	2.3 ± 0.4 (27%)	2.1 ± 0.5 (34%)
Fe_total_ mg L^−1^	100 ± 40	210 ± 30

* Removal percentages are included in brackets.

**Table 3:** Summary of results of the UVA-LED photo-Fenton treatment of mature LL pre-treated by an optimal electro-coagulation followed by an optimized conventional coagulation pre-treatment combination.

	Raw LL	UVA-LED Photo-Fenton [H_2_O_2_]:COD = 2.125, 120 min	UVA-LED Photo-Fenton [H_2_O_2_]:COD = 1.063, 45 min
pH	8.1 ± 0.1	4.4 ± 0.1	3.9 ± 0.1
Conductivity mS cm^−1^	20.1 ± 0.8	17.1 ± 0.8 (15%) *	16.7 ± 0.8 (17%)
Color mg Pt L^−1^	19,600 ± 1200	90 ± 20 (99%)	120 ± 30 (99%)
COD mg O_2_ L^−1^	5025 ± 500	290 ± 30 (94%)	453 ± 50 (89%)
SUVA L mg^−1^ m^−1^	3.1 ± 0.4	1.0 ± 0.2 (67%)	1.2 ± 0.3 (60%)
Fe_total_ mg L^−1^	4.5 ± 1	210 ± 30	210 ± 30
BOD_5_/COD	0.01 ± 0.01	0.40 ± 0.10	0.40 ± 0.10

* Removal percentages are included in brackets.

**Table 4:** Treatment cost assessment of the individual and global processes included in the herein assayed optimized treatment combination strategy for landfill leachate.

**EC Costs**	**EC** (120 min; pH = 5; 10 mA cm^−2^)	
H_2_SO_4_, EUR m^−3^	0.95 (7.3 g L^−1^)	
Iron electrode, EUR m^−3^	1.00 (2.0 g L^−1^)	
EC power consumption, EUR m^−3^	0.75	
EC total cost, EUR m^−3^	2.70	
EC total cost, EUR kg^−1^ of COD removed	0.86	
**Conventional coagulation (CC) costs**	**CC** (pH = 6; 1 g L^−1^ FeCl_3_∙6H_2_O)	
CC. Total cost, EUR m^−3^	0.20	
CC. Total cost, EUR kg^−1^ of COD	0.24	
**Oxidation costs**	**UVA-LED photo-Fenton [H_2_O_2_]:COD = 2.125, 120 min**	**UVA-LED photo-Fenton** **[H_2_O_2_]:COD = 1.063, 45 min**
H_2_O_2_, EUR m^−3^	1.90 (1.81 g L^−1^)	0.95 (0.91 g L^−1^)
Oxidation power consumption, EUR m^−3^	8.80	3.30
Oxidation total cost, EUR m^−3^	10.70	4.25
Oxidation total cost, EUR kg^−1^ of removed COD	19.11	10.63
**Sludge management**, EUR m^−3^	8.50	8.50
**Total process cost**, EUR m^−3^	22.10	15.65
**Total process cost**, EUR kg^−1^ of removed COD	5.05	3.42

Results expressed per m^3^ refer to the volume of treated landfill leachate and results expressed per kg of removed COD refers to the specific amount of COD removed in each treatment step.

**Table 5:** Comparison of treatment cost (excluding sludge management) and final COD result among different pre-treatment alternatives for the photo-Fenton treatment ([H_2_O_2_]:COD = 1.063) of similar mature landfill leachate samples (COD ≈ 5000 mg L^−1^).

	Total Treatment Cost EUR kg^−1^ of Removed COD	Final COD mg O_2_ L^−1^
EC + CC + UVA-LED	1.57	450
CC + Hg 450 W photo-Fenton [11]	1.56	826
EC + UVA-LED photo-Fenton [30]	3.48	1000
EC + Conventional Fenton [30] UVA-LED photo-Fenton	1.09 3.83	1000 2000

## Data Availability

Not applicable.

## Sample Availability

Samples of the compounds are available from the authors.

## References

[1] Iskander M.S., Zhao R., Pathak A., Gupta A., Pruden A., Novak J.T., He Z.A. (2018). review of landfill leachate induced ultraviolet quenching substances: Sources, characteristics, and treatment. Water Res..

[2] Kaza S., Yao L., Bhada-Tata P., Van Woerden F. (2018). What a Waste 2.0: A Global Snapshot of Solid Waste Management to 2050.

[3] Deng Y., Englehardt J.D. (2006). Treatment of landfill leachate by the Fenton process. Water Res..

[4] Chou J.D., Wey M.Y., Liang H.H., Chang S.H. (2009). Biotoxicity evaluation of fly ash and bottom ash from different municipal solid waste incinerators. J. Hazard. Mater..

[5] Dastjerdi B., Strezov V., Kumar R., He J., Behnia M. (2021). Comparative life cycle assessment of system solution scenarios for residual municipal solid waste management in NSW. Aust. Sci. Total Environ..

[6] Abbas A.A., Jingsong G., Ping L.Z., Ya P.Y., Al-Rekabi W.S. (2009). Review on LandWll Leachate Treatments. Res. J. Appl. Sci..

[7] Mukherjee S., Mukhopadhyay S., Hashim M.A., Sen Gupta B. (2015). Contemporary environmental issues of landfill leachate: Assessment and remedies. Crit. Rev. Environ. Sci. Technol..

[8] Ghosh P., Thakur L.S., Kaushik A. (2017). Bioassays for toxicological risk assessment of landfill leachate: A review. Ecotoxicol. Environ. Saf..

[9] Zolfaghari M., Jardak K., Drogui P., Brar S.K., Buelna G., Dubé R. (2016). Landfill leachate treatment by sequential membrane bioreactor and electro-oxidation processes. J. Environ. Manag..

[10] Foo K.Y., Hameed B.H. (2009). An overview of landfill leachate treatment via activated carbon adsorption process. J. Hazard. Mater..

[11] Tejera J., Miranda R., Hermosilla D., Urra I., Negro C., Blanco A. (2019). Treatment of a Mature Landfill Leachate: Comparison between Homogeneous and Heterogeneous Photo-Fenton with Different Pretreatments. Water.

[12] Ahmed F.N., Lan C.Q. (2012). Treatment of landfill leachate using membrane bioreactors: A review. Desalination.

[13] Kjeldsen P., Barlaz M.A., Rooker A.P., Baun A., Ledin A., Christensen T.H. (2002). Present and long-term composition of MSW landfill leachate: A review. Crit. Rev. Environ. Sci. Technol..

[14] Luo H., Zeng Y., Cheng Y., He D., Pan X. (2020). Recent advances in municipal landfill leachate: A review focusing on its characteristics, treatment, and toxicity assessment. Sci. Total Environ..

[15] Wu C., Chen W., Gu Z., Li Q. (2020). A review of the characteristics of Fenton and ozonation systems in landfill leachate treatment. Sci. Total Environ..

[16] Deng Y., Zhu X., Chen N., Feng C., Wang H., Kuang P., Hu W. (2020). Review on electrochemical system for landfill leachate treatment: Performance, mechanism, application, shortcoming, and improvement scheme. Sci. Total Environ..

[17] Boumechhour F., Rabah K., Lamine C., Said B.M. (2013). Treatment of landfill leachate using Fenton process and coagulation/flocculation. Water Environ. J..

[18] Amor C., De Torres-Socías E., Peres J.A., Maldonado M.A., Oller I., Malato S., Lucas M.S. (2015). Mature landfill leachate treatment by coagulation/flocculation combined with Fenton and solar photo-Fenton processes. J. Hazard. Mater..

[19] Rebolledo L.P., Arana V.A., Trilleras J., Barros G.E., González-Solano A.J., Maury-Ardila H. (2019). Efficiency of Combined Processes Coagulation/Solar Photo Fenton in the Treatment of Landfill Leachate. Water.

[20] Jiang J.Q. (2015). The role of coagulation in water treatment. Curr. Opin. Chem. Eng..

[21] Litter M.I., Slodowicz M. (2017). An overview on heterogeneous Fenton and photoFenton reactions using zerovalent iron materials. J. Adv. Oxid. Technol..

[22] Zhang M.H., Dong H., Zhao L., Wang D.X., Meng D. (2019). A review on Fenton process for organic wastewater treatment based on optimization perspective. Sci. Total Environ..

[23] Asaithambi P., Govindarajan R., Yesuf M.B., Selvakumar P., Alemayehu E. (2020). Enhanced treatment of landfill leachate wastewater using sono (US)-ozone (O3)–electrocoagulation (EC) process: Role of process parameters on color, COD and electrical energy consumption. Process Saf. Environ..

[24] Djelal H., Lelievre Y., Ricordel C. (2015). Combination of Electro-Coagulation and biological treatment by bioaugmentation for landfill leachate. Desalin. Water Treat..

[25] Le S.T., Le K.C. (2019). Reduction of COD in Nam Son landfill leachate by electro-Fenton as secondary treatment after electrocoagulation pretreatment. Vietnam J. Sci. Technol..

[26] Nidheesh P.V., Scaria J., Babu D.S., Kumar M.S. (2020). An overview on combined electrocoagulation/degradation processes for the effective treatment of water and wastewater. Chemosphere.

[27] Phalakornkule C., Mangmeemak J., Intrachod K., Nuntakumjorn B. (2010). Pretreatment of palm oil mill effluent by electrocoagulation and coagulation. ScienceAsia.

[28] Bazrafshan E., Mostafapour F.K., Farzadkia M., Ownagh K.A., Mahvi A.H. (2012). Slaughterhouse wastewater treatment by combined chemical coagulation and electrocoagulation process. PLoS ONE.

[29] Bazrafshan E., Alipour M.R., Mahvi A.H. (2016). Textile wastewater treatment by application of combined chemical coagulation, electrocoagulation, and adsorption processes. Desalin. Water Treat..

[30] Tejera J., Hermosilla D., Gascó A., Miranda R., Alonso V., Negro C., Blanco A. (2021). Treatment of mature landfill leachate by electrocoagulation followed by Fenton or UVA-LED photo-Fenton processes. J. Taiwan Inst. Chem. Eng..

[31] Kim S.M., Geissen S.U., Vogelpohl A. (1997). Landfill leachate treatment by a photoassisted Fenton reaction. Water Sci. Technol..

[32] Montalti M., Credi A., Prodi L., Gandolfi M.T. (2006). Handbook of Photochemistry.

[33] Hatchard C., Parker C.A. (1956). Mathematical and Physical Sciences. P. Roy. Soc. Lond. A Mat..

[34] APHA A. (2005). Standard Methods for the Examination of Water and Wastewater.

[35] Barndõk H., Merayo N., Blanco L., Hermosilla D., Blanco A. (2016). Application of on-line FTIR methodology to study the mechanisms of heterogeneous advanced oxidation processes. Appl. Catal. B.

[36] Bacardit J., Stötzner J., Chamarro E., Esplugas S. (2007). Effect of salinity on the photo-Fenton process. Ind. Eng. Chem. Res..

[37] Hermosilla D., Cortijo M., Huang C.P. (2009). Optimizing the treatment of landfill leachate by conventional Fenton and photo-Fenton processes. Sci. Total Environ..

[38] Ertugay N., Kocakaplan N., Malkoc E. Investigation of pH Effect with Fenton-Like Oxidation using ZVI in Treatment of the Landfill Leachate. Proceedings of the 16th International Symposium on Environmental Issues and Waste Management in Energy and Mineral Production.

[39] Dia O., Drogui P., Buelna G., Dubé R., Ihsen B.S. (2017). Electrocoagulation of bio-filtrated landfill leachate: Fractionation of organic matter and influence of anode materials. Chemosphere.

[40] Gomes A.I., Santos S.G.S., Silva T.F.C.V., Boaventura R.A.R., Vilar V.J.P. (2019). Treatment train for mature landfill leachates: Optimization studies. Sci. Total Environ..

[41] Dogaris I., Ammar E., Philippidis G.P. (2020). Prospects of integrating algae technologies into landfill leachate treatment. World J. Microbiol. Biotechnol..

[42] Tejera J., Hermosilla D., Miranda R., Gascó A., Alonso V., Negro C., Blanco A. (2020). Assessing an integral treatment for landfill leachate reverse osmosis concentrate. Catalysts.

[43] Blanco L., Hermosilla D., Merayo N., Blanco A. (2016). Assessing the use of zero-valent iron microspheres to catalyze Fenton treatment processes. J. Taiwan Inst. Chem. Eng..

